# Reports of Cases Treated at the Men’s Lunatic Asylum, Attached to the Philadelphia Hospital, Blockley, *during the Years* 1836, 1837, 1838

**Published:** 1839-01-26

**Authors:** W. W. Gerhard

**Affiliations:** Attending Physician


### REPORTS OF CASES TREATED AT THE MEN'S LUNATIC ASYLUM, ATTACHED TO THE PHILADELPHIA HOSPITAL, BLOCKLEY,

*During the Years* 1836, 1837, 1838.

By W. W. Gerhard, M. D., Attending Physician.

*Statement of* 376 *Male Patients admitted into the Lunatic Asylum, Blockley Hospital, for the year commencing January* 1*st*, 1836, *to January* 1*st*, 1837, *viz.*:

| DIAGNOSIS. | *No. of Cases.* | 10 to 20 | 20 to 30 | 30 to 40 | 40 to 50 | 50 to 60 | 60 to 70 | 70 to 80 | 80 to 90 | 90 to 100 | *Result.* | *Cured.* | *Died.* | *Remaining.* | *Total.* |
|---|---|---|---|---|---|---|---|---|---|---|---|---|---|---|---|
| Mania-a-Potu, White, . . . . | 204 | 1 | 32 | 78 | 55 | 32 | 5 | 0 | 1 | 0 | " | 174 | 29 | 1 | 204 |
| Mania-a-Potu, Black, . . . . | 7 | 0 | 1 | 4 | 1 | 0 | 0 | 1 | 0 | 0 | " | 5 | 2 | 0 | 7 |
| Insane, White, . . . . . . . | 58 | 1 | 20 | 18 | 10 | 3 | 3 | 3 | 0 | 0 | " | 37 | 8 | 13 | 58 |
| Insane, Black, . . . . . . . | 16 | 1 | 8 | 5 | 1 | 0 | 0 | 0 | 1 | 0 | " | 10 | 3 | 3 | 16 |
| Intemperate, White, . . . . . | 88 | 1 | 17 | 23 | 25 | 13 | 8 | 1 | 0 | 0 | " | 82 | 5 | 1 | 88 |
| Intemperate, Black, . . . . . | 3 | 0 | 1 | 2 | 0 | 0 | 0 | 0 | 0 | 0 | " | 3 | 0 | 0 | 3 |
| Total, | 376 | 4 | 79 | 130 | 92 | 48 | 16 | 5 | 2 | 0 | " | 311 | 47 | 18 | 376 |

*Statement of* 363 *Male Patients admitted into the Lunatic Asylum, Blockley Hospital, for the year commencing January* 1*st*, 1837, *to January* 1*st*, 1838, *viz.:*

| DIAGNOSIS. | *No. of Cases.* | 10 to 20 | 20 to 30 | 30 to 40 | 40 to 50 | 50 to 60 | 60 to 70 | 70 to 80 | 80 to 90 | 90 to 100 | *Result.* | *Cured.* | *Died.* | *Remaining.* | *Total.* |
|---|---|---|---|---|---|---|---|---|---|---|---|---|---|---|---|
| Mania-a-Potu, White, . . . . | 184 | 0 | 22 | 65 | 60 | 27 | 10 | 0 | 0 | 0 | " | 157 | 26 | 1 | 184 |
| Mania-a-Potu, Black, . . . . | 6 | 0 | 1 | 3 | 1 | 0 | 1 | 0 | 0 | 0 | " | 5 | 1 | 0 | 6 |
| Insane, White, . . . . . . . | 78 | 7 | 21 | 19 | 18 | 5 | 8 | 0 | 0 | 0 | " | 44 | 8 | 26 | 78 |
| Insane, Black, . . . . . . . | 21 | 1 | 11 | 3 | 3 | 3 | 0 | 0 | 0 | 0 | " | 7 | 1 | 13 | 21 |
| Intemperate, White, . . . . . | 69 | 0 | 16 | 26 | 15 | 9 | 3 | 0 | 0 | 0 | " | 61 | 2 | 6 | 69 |
| Intemperate, Black, . . . . . | 5 | 0 | 3 | 0 | 2 | 0 | 0 | 0 | 0 | 0 | " | 5 | 0 | 0 | 5 |
| Total, | 363 | 8 | 74 | 116 | 99 | 44 | 22 | 0 | 0 | 0 | " | 279 | 38 | 46 | 363 |

*Statement of* 322 *Male Patients admitted into the Lunatic Asylum, Blockley Hospital, for the year commencing January* 1*st*, 1838, *to January* 1*st*, 1839, *viz.:*

| DIAGNOSIS. | *No. of Cases.* | 10 to 20 | 20 to 30 | 30 to 40 | 40 to 50 | 50 to 60 | 60 to 70 | 70 to 80 | 80 to 90 | 90 to 100 | *Result.* | *Cured.* | *Died.* | *Remaining.* | *Total.* |
|---|---|---|---|---|---|---|---|---|---|---|---|---|---|---|---|
| Mania-a-Potu, White,* . . . . | 120 | 20 | 41 | 38 | 17 | 4 | 0 | 0 | 0 | 0 | " | 106 | 14 | 0 | 120 |
| Mania-a-Potu, Black, . . . . | 2 | 0 | 1 | 1 | 0 | 0 | 0 | 0 | 0 | 0 | " | 2 | 0 | 0 | 2 |
| Intemperate, White, . . . . . | 102 | 9 | 51 | 27 | 13 | 2 | 0 | 0 | 0 | 0 | " | 96 | 4 | 2 | 102 |
| Intemperate, Black, . . . . . | 10 | 3 | 3 | 3 | 1 | 0 | 0 | 0 | 0 | 0 | " | 9 | 1 | 0 | 10 |
| Insane, White, . . . . . . . | 76 | 6 | 20 | 26 | 14 | 9 | 1 | 0 | 0 | 0 | " | 52 | 2 | 22 | 76 |
| Insane, Black, . . . . . . . | 12 | 3 | 5 | 3 | 0 | 0 | 1 | 0 | 0 | 0 | " | 7 | 1 | 4 | 12 |
| Total, | 322 | 41 | 121 | 98 | 45 | 15 | 2 | 0 | 0 | 0 | " | 272 | 22 | 28 | 322 |

* There is an error in this line. The number of patients should be 118, and of deaths 12. The error arose from the circumstance that two patients were admitted in a state of partial insensibility, and could not be removed to the proper ward. They died, one from the effects of cold, while in a state of intoxication, and the other from apoplexy.

From these tables, it appears that the proportion of recoveries is as follows:

In the year 1836, . . 63½ per cent.

" 1837, . . 51½ "

" 1838, . . 67⅘ "

The tables include all cases of insanity admitted into the Men's Lunatic Asylum of the Philadelphia Hospital, (attached to the almshouse.) There are no separate tables for the acute and chronic cases, on account of an entire want of the necessary information respecting the previous history of a large proportion of the patients. Many of them were not sent to the hospital by their friends, but by strangers, who were altogether ignorant of the early symptoms of their disease.

The tables are also defective, from the absence of a sufficiently accurate classification. They are therefore altogether omitted.

The proportion of chronic, that is, of unfavourable cases, is undoubtedly very large; quite as large, in all probability, as in any similar institution, for no patient is excluded from the supposed incurability of his disease.

The patients may be divided into several classes. First. Those brought to the hospital by their friends, many of whom enter at an early period of the disease. Secondly. Individuals sent by the police, on account of the obvious derangement under which they labour. Thirdly. Patients discharged from the Pennsylvania Hospital, who have not recovered within the time for which they were admitted. Fourthly. Men who have been discharged from prison, in a state of insanity, after the expiration of the period for which they had been confined.

This enumeration shows that the patients are not, as a class, amongst the most promising; and we have every reason to believe that the proportion of acute cases is not greater than in other institutions of the same kind. With a few insignificant exceptions, the admissions were confined to what is strictly to be termed insanity. The exceptions were cases of cerebral disease and of delirium, which are not considered of the same nature with insanity, properly so called, but are so closely allied to it, as to require seclusion. These cases are always to be found in insane hospitals, and constitute a certain proportion of those which are classed under the title of acute insanity. These diseases are not more curable than insanity, and do not give a more favourable appearance to a report, unless they should be so numerous as greatly to increase the proportion of acute cases. For, in all well regulated hospitals, acute insanity is one of the most curable of diseases.

It will be seen by a reference to the tables, that the proportion of recoveries varies but little in the years 1836 and 1838, while 1837 falls much below the average of the other two years. It is not necessary, for our purpose, to inquire into the causes which rendered the success in the year 1837 less than in the other years; those which appeared most efficient, were unavoidable.

Delirium tremens and intemperance are classed under separate heads. The division is not strictly logical, as many of the cases of intemperance passed into decided delirium tremens, and several terminated fatally in each year, either from the occurrence of delirium tremens, or some other disease not directly connected with the habits of the patients.

The success in the management of the insane, it will be seen, is highly gratifying, and sustains comparison with institutions placed under much more favourable circumstances. Many obstacles have been removed by the care and exertions of the managers of the hospital, but others were inseparable from its position, and could only be surmounted by much patient perseverance.—A large portion of the success of the treatment is certainly owing to the humane and constant attention of Mr. Gillespie, the superintendent of the Lunatic Asylum.

There are undoubtedly some errors in the tables, and probably not a few cases are considered as cured, who, in reality, were not completely restored to usefulness. The same difficulty, however, occurs in the reports of all institutions for the insane. A proportion of inmates are curable only to a certain extent; still, inasmuch as their mind has recovered the full degree of strength of which it is susceptible, they are regarded as examples of recovery. There are several circumstances, however, which give a more unfavourable appearance to the table, than is offered in the reports of institutions into which the wealthier classes of society are admitted. Patients who are slightly relieved, are usually allowed to remain amongst the chronic lunatics, and are very rarely removed by their friends.

The Philadelphia Hospital is not designed for lunatics. They are admitted into the wings of the building, which is chiefly appropriated to the sick. It does not, therefore, offer the advantages that are presented by institutions of moderate size, which are specially adapted to the treatment of the insane. It is, however, an error to suppose that the proportion of recoveries, taking acute and chronic cases together, would be much greater at a special hospital, for the plain reason, that chronic patients constitute but a very small percentage of successful cases. The advantages which are presented by an institution constructed expressly for the insane, may be classed under two heads: First, the condition of the incurable may be greatly ameliorated; secondly, facilities are afforded for the application of moral treatment, which occasionally proves successful in some cases of chronic insanity, which cannot be successfully treated except in institutions exclusively destined for the insane.

